# Both Maturation and Survival of Human Dendritic Cells are Impaired in the Presence of Anergic/Suppressor T Cells

**DOI:** 10.1080/10446670310001593505

**Published:** 2003-03

**Authors:** L. Frasca, C. Scottà, G. Lombardi, E. Piccolella

**Affiliations:** 1 Department of Cell Development and Biology “La Sapienza” University Via dei Sardi Rome 70 - 00185 Italy; 2 Department of Immunology Imperial College of Medicine Hammersmith Hospital London W12 0NN UK

## Abstract

T cell suppression is a well established phenomenon, but the mechanisms involved are still a matter of debate. Mouse anergic T cells were shown to suppress responder T cell activation by inhibiting the antigen presenting function of DC. In the present work we studied the effects of co-culturing human anergic CD4^+^ T cells with autologous dendritic cells (DC) at different stages of maturation. Either DC maturation or survival, depending on whether immature or mature DC where used as APC, was impaired in the presence of anergic cells. Indeed, MHC and costimulatory molecule up-regulation was inhibited in immature DC, whereas apoptotic phenomena were favored in mature DC and consequently in responder T cells. Defective ligation of CD40 by CD40L (CD154) was responsible for CD95-mediated and spontaneous apoptosis of DC as well as for a failure of their maturation process. These findings indicate that lack of activation of CD40 on DC by CD40L-defective anergic cells might be the primary event involved in T cell suppression and support the role of CD40 signaling in regulating both activation and survival of DC.


*Clinical & Developmental Immunology*, March 2003, Vol. 10 (1), pp. 61–65

Taylor & Francis healthsciences

### Both Maturation and Survival of Human Dendritic Cells are Impaired in the Presence of Anergic/Suppressor T Cells

L. FRASCA[a,*], C. SCOTTÀ[a], G. LOMBARDI[b] and E. PICCOLELLA[a]

[a]*Department of Cell Development and Biology, "La Sapienza" University, Via dei Sardi, 70 - 00185, Rome, Italy;* [b]*Department of Immunology, Imperial College of Medicine, Hammersmith Hospital, W12 0NN, London, UK*

T cell suppression is a well established phenomenon, but the mechanisms involved are still a matter of debate. Mouse anergic T cells were shown to suppress responder T cell activation by inhibiting the antigen presenting function of DC. In the present work we studied the effects of co-culturing human anergic CD4$^{+}$ T cells with autologous dendritic cells (DC) at different stages of maturation. Either DC maturation or survival, depending on whether immature or mature DC where used as APC, was impaired in the presence of anergic cells. Indeed, MHC and costimulatory molecule up-regulation was inhibited in immature DC, whereas apoptotic phenomena were favored in mature DC and consequently in responder T cells. Defective ligation of CD40 by CD40L (CD154) was responsible for CD95-mediated and spontaneous apoptosis of DC as well as for a failure of their maturation process. These findings indicate that lack of activation of CD40 on DC by CD40L-defective anergic cells might be the primary event involved in T cell suppression and support the role of CD40 signaling in regulating both activation and survival of DC.

*Keywords*: Anergy; CD40–CD40L interaction; DC; T cell suppression

### INTRODUCTION

T cells can transfer tolerance from a tolerant to a naïve host (Gerson, 1975; Charlton *et al.*, 1994) a phenomenon referred to as "infectious tolerance" (Zhai and Kupie-Weglinski, 1999). We have shown that human CD4$^{+}$ T cells, rendered tolerant, exert suppressive activity on responder T cells *in vitro* (Lombardi *et al.*, 1994; Frasca *et al.*, 1997). To assess whether suppression was mediated through the APC as shown in mice (Vendetti *et al.*, 2000), here we analysed the susceptibility of both immature and mature human DC to suppressive signals mediated by CD4$^{+}$ T cell clones anergized with OKT3 mAb (Frasca *et al.*, 1997). Moreover, we followed the fate of responder cells cultured with these DC in the presence of anergic cells.

### RESULTS

### Anergic T Cells Exert Suppression in the Presence of both Immature and Mature DC

We have demonstrated previously that human anergic CD4$^{+}$ T cells suppress proliferation of responder T cells to EBV-B transformed B cell lines (B-LCL) presenting antigen to both cell types (Lombardi *et al.*, 1994; Frasca *et al.*, 1997). Here we wanted to analyze the same phenomenon in a more physiological condition using DC as APC. Clone F17, HA307-19-specific and DRB1*1101-restricted, was in part either anergized (Frasca *et al.*, 1997) or activated with PMA + I. Figure 1 shows its suppressive activity on responder cells of the same clone (anergic/responder cells ratio 3:1), in the presence of both immature and mature DRB1*1101$^{+}$ HA307-19-pulsed DC. Data are expressed as percentage of suppression of responder cells proliferation. It is clear that anergic cells exert suppression independently on the maturational stages of DC. As control, T cells activated with PMA + I did not affect responder cell proliferation. To learn about the mechanism of suppression, we first evaluated expression of HLA-DR and CD86 molecules on DC pulsed with HA307-19 and cultured with either anergic or activated F17. In fact, we wanted to verify a possible inhibitory effect of anergic cells on the stimulatory capacity of DC. Figure 2a shows that culture with activated cells (and also responder cells, not shown) increased the percentage of these molecules expression on immature (but not mature) DC after 48 h of co-culture, as measured by FACS. In contrast, culture with anergic cells did not. Of note, no modification of these molecules expression was observed when mature DC were used in the experiment. This result suggested that, in the presence

*Corresponding author. Tel.: +39-06-49917584/6. Fax: +39-06-49917594. E-mail: enza.piccolella@uniroma1.it

ISSN 1740-2522 print/ISSN 1740-2530 online © 2003 Taylor & Francis Ltd
DOI: 10.1080/10446670310001593505

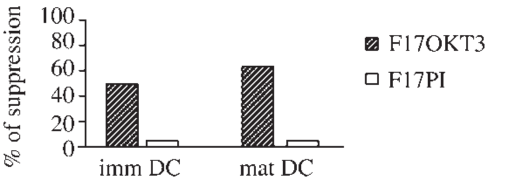

FIGURE 1 Anergic T cells inhibit T cell proliferation induced by "mature" and "immature" DC. Responder F17 ($5 \times 10^3$) was cultured for 72 h with $5 \times 10^3$ DRB1*1101$^+$ "mature" or "immature" DC pulsed with HA307-19 in the absence or in the presence of $1.5 \times 10^4$ either anergic (F17OKT3) or PI-activated F17 (F17PI). The results obtained in a proliferation assay, are expressed as percent of inhibition of responder F17 proliferation and derived by one representative experiment.

of immature DC, anergic cells could mediate suppression by antagonizing maturating stimuli provided by responder cells. To verify this, induction of CD86 up-regulation by responder F17 was assessed in the presence or absence of anergic cells (Fig. 2b). When anergic cells were present in the culture together with responder cells CD86 up-regulation in DC was strongly reduced showing that our hypothesis was correct. However, we wonder how anergic cells could exert suppression in the presence of fully mature DC that did not modify their phenotype in any condition (Fig. 2a). Since in our hands mature DC expressed high amount of CD95, unlikely the immature ones and anergic T cells expressed CD95L (data not shown) we verified the possible implication of CD95-mediated apoptosis in suppression. We first assessed the ability of anergic cells to inhibit proliferation of responder cells after incubation of either responder T cells or DC,

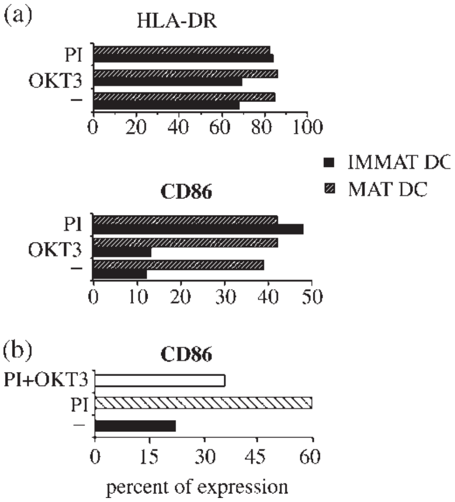

FIGURE 2 Anergic T cells fail to upregulate MHC class II and CD86 molecule expression on "immature" DC cells and inhibit CD86 upregulation induced by responder cells. (a) $2 \times 10^4$ "immature" or "mature" DRB1*1101$^+$ DC, pulsed with HA307-19, were cultured either alone (−) or with activated F17 (PI) or with F17 anergized on immobilized OKT3 antibody (OKT3) at 37°C for 48 h. (b) $4 \times 10^4$ "immature" DRB1*1101$^+$ DC pulsed with HA307-19 were cultured either alone (−) or with activated F17 (PI) or both activated and anergic F17 (PI + OKT3) (anergic or activated/anergic T cell ratio 3:1) for 48 h. Data in (a) and (b), shown as percent of expression of each marker measured by FACS, are from one of two separate experiments.

separately, with the antagonistic anti-CD95 antibody M3. When either DC or responder T cells were treated (Fig. 3a) suppression was reduced. To confirm these data we performed a cytotoxic assay in which all three cells types (responder cells, anergic cells, and mature DC) were present in the same culture at the ratio utilized in proliferation experiments. Anergic cells were the effectors in the assay and we marked with $^{51}$Cr either only responder T cells or DC in the same well. We also assessed direct killing of target DC or responder cells alone. The results in Fig. 3b show that DC and responder cells were killed in a significant fashion in the presence of anergic cells when all three-cell types were present in the same culture. Death was mainly mediated *via* CD95-CD95L. In contrast, the capacity of anergic cells to kill separately responder cells or mature DC was different. Only DC were significantly killed in this condition. We interpret this as an indication that anergic cells kill not only the APC, but also responder cells, only when the three cell types come into contact by interactions driven by antigen recognition.

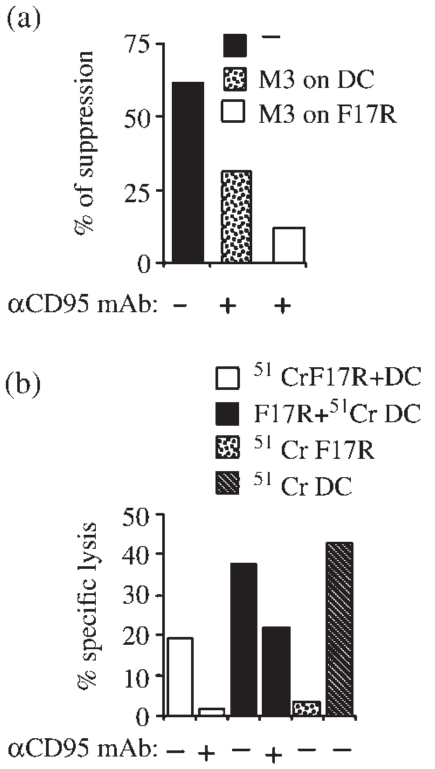

FIGURE 3 Anergic T cells kill both DC and responder T cells, mainly through the CD95-CD95L system. (a) $5 \times 10^3$ responder F17 cells were cultured with the specific DC in the absence or in the presence of anergic T cells at anergic/responder cells ratio 3:1. Responder cells and DC cultured in the presence of anergic cells were untreated (−) or either responder cells (M3 on F17R) or DC (M3 on DC) were pre-treated with 5 μg/ml of antagonistic anti-CD95 antibody (M3). Results are expressed as in Fig. 1. (b) Effector $9 \times 10^3$ F17 anergic T cells were cultured with $3 \times 10^3$ DRB1*1101$^+$ unlabeled DC + $3 \times 10^3$ $^{51}$Cr-labeled responder F17 ($^{51}$Cr F17R + DC) or with $3 \times 10^3$ $^{51}$Cr-labeled DC + $3 \times 10^3$ unlabeled F17 (F17R + $^{51}$Cr DC) or with $3 \times 10^3$ responder F17 ($^{51}$Cr F17R) or DC ($^{51}$Cr DC) alone. The results, expressed as percentage of specific lysis, are representative of two experiments.

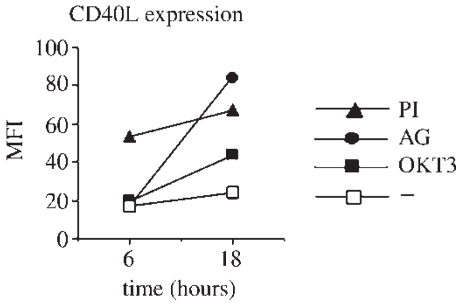

FIGURE 4 Anergic CD4$^+$ T cells show impaired expression of CD40L. $2 \times 10^5$ cells of F17 clone were either untreated (−) or anergized with immobilized OKT3 (OKT3) or stimulated with $3 \times 10^5$ HLA-DRB1*1101$^+$ B-LCL pre-pulsed o.n. with 10 μg/ml of HA307-19 (Ag) or with PMA + I (PI) in 48-well plates. Cells were cultured at 37°C and CD40L expression was assessed by staining with PE-conjugated anti-human CD40L mAb (TRAP-1). The results are expressed as mean of fluorescence intensity (MFI) measured by FACS and derived from one of four separate experiments.

### Human Anergic CD4$^+$ T Cells Show Defective Up-regulation of CD40L

To understand the reason for lack of maturation of immature DC and increased apoptosis of mature DC in the presence of anergic cells we measured CD40L expression by anergic cells. We looked at CD40L because tolerant T cells from mice were described to express altered level of this molecule (Bowen *et al.*, 1995). Moreover, it has been shown that engagement of CD40 by CD40L prevents both spontaneous and CD95-induced cell death of DC (Ludewig *et al.*, 1995; Bjorck *et al.*, 1997; Koppi *et al.*, 1997) and activates immature DC, a phenomenon described as APC "conditioning" or "licensing" (Lanzavecchia, 1998). Figure 4 shows the kinetic of CD40L expression on clone F17 following activation with PMA + I or with DRB1*1101$^+$ B-LCL pulsed with HA307-19, and after anergy induction expressed as mean of fluorescence intensity (MFI) measured by FACS. T cells receiving activating stimuli significantly up-regulated CD40L, whereas anergic cells did not. To produce functional data, we activated CD40 molecules on target DC by a cross-linked anti-CD40 mAb (M.M.) and used again anergic F17 as effector in a cytotoxic assay. As an important control, we tested the capacity of PI-activated cells, unable to exert suppression in Fig. 1, to kill DC. These cells also express CD95L (a typical activation marker, not shown) together with high amount of CD40L, unlikely anergic cells shown to be CD95L$^+$ in the absence of CD40L up-regulation, and they should rescue DC from apoptosis. Indeed, the results in Fig. 5a confirm the expectation that the killing capacity of high CD40L-expressing activated T cells against target DC was lower than that of anergic cells (although also mainly dependent on CD95-pathway, not shown). Moreover, in the same experiment we observed inhibition of apoptosis of DC activated through CD40. This strongly supported the idea that increased death of DC, in the presence of anergic cells, was due to lack of CD40 engagement on these APC.

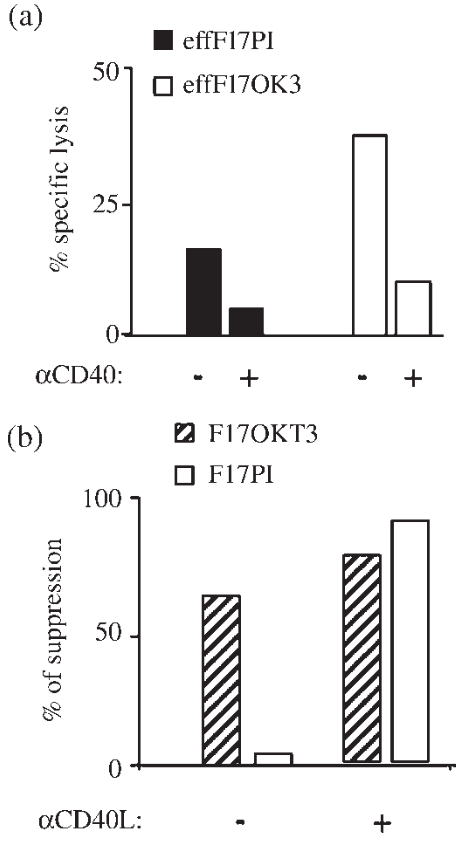

FIGURE 5 CD40/CD40L interaction protects DC from death and treatment of activated T cells with anti-CD40L antagonistic antibody favors suppressive phenomena. (a) Either $9 \times 10^3$ effector anergic (effF17OKT3) or PI-activated (effF17PI) F17 cells cultured with $3 \times 10^3$ HA307-19-pulsed $^{51}$Cr-labeled DRB1*1101$^+$ "mature" DC either untreated or treated with a cross-linked anti-CD40 mAb (G28-5) as indicated. The results are expressed as the percentage of specific $^{51}$Cr release and are representative of two experiments. (b) Responder F17 ($5 \times 10^3$ cells) was cultured with $5 \times 10^3$ DRB1*1101$^+$ "mature" DC pulsed with HA307-19 either alone or in the presence of $1.5 \times 10^4$ F17 anergic cells (F17OKT3) or in the presence of $1.5 \times 10^4$ PMA + I stimulated F17 T cells (F17PI) either untreated or treated with the antagonistic anti-CD40L antibody as indicated. Data are reported as in Fig. 1.

We reasoned that if this hypothesis was correct, we should observe suppressive phenomena in the presence not only of anergic but also activated cells when CD40–CD40L interaction is interrupted during T-DC contact. To verify this, we pre-treated both activated and anergic cells with an antagonistic anti-CD40L mAb and repeated suppression experiments. As expected, in these conditions also activated cells could exert suppression (Fig. 5b) confirming that by disrupting CD40–CD40L interaction protection of DC from death during cognate interaction with CD95L$^+$ T cells was abolished. To confirm that poor CD40L expression by anergic cells was insufficient for a productive interaction with CD40 on DC we decided to look at Bcl-2 expression in DC cultured with either activated or anergic cells. CD40 engagement produces up-regulation of this anti-apoptotic molecule (Bjorck *et al.*, 1997; Koppi *et al.*, 1997) mainly implicated in protection

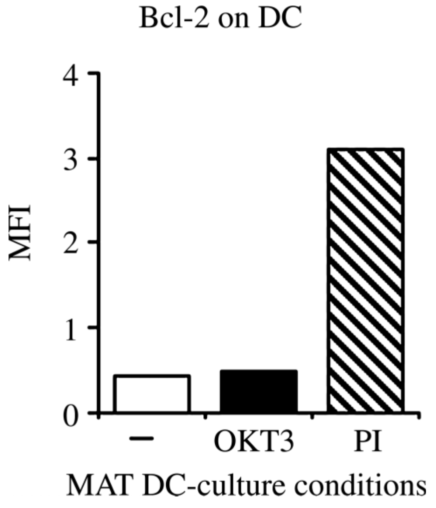

FIGURE 6 Anergic cells do not favor up-regulation of Bcl-2 in DC. $2 \times 10^4$ "mature" DRB1*1101$^+$ DC, pulsed with HA307-19, were cultured either alone (−) or with anergic (OKT3) or activated (PI) F17. The cells were co-cultured at 37°C for 48 h and Bcl-2 expression on DC was assessed by FACS after intracellular staining. Data shown, expressed as mean of fluorescence intensity (MFI), are from one of two separate experiments.

from spontaneous apoptosis of DC (its role in CD95-mediated apoptosis is still controversial). Following intracellular staining of DC cultured for 24 h with anergic or activated F17 (and responder F17, not shown) we found that (Fig. 6) anergic cells were unable to induce Bcl-2 up-regulation as compared to non-anergic cells. Of note, we observed a reduction of more than 50% of spontaneous apoptosis of DC only in the presence of activated, but not anergic cells (data not shown) after 24 and 48 h of co-culture.

### DISCUSSION

The principal novelty of our work is that human anergic T cells can always suppress the APC function of DC: (i) by inhibiting their maturation process (ii) by triggering apoptotic pathways in fully mature DC.

Our results reinforce the important role of CD40 as regulatory molecule of DC function and demonstrate that CD40 engagement on DC can be modulated by regulation of its natural ligand expression on helper T cells. Although DC can receive maturating stimuli from pathogens, it is well known that helper T cells exert a predominant role in the activation of these APC (a phenomenon described as licensing or conditioning, Lanzavecchia, 1998). Therefore the induction of CD40L-defective anergic cells can be a mechanism that by impairing T cell help, eventually lead to inhibition of immunity. Moreover, since the same interaction also regulates CD95-mediated apoptotic pathways in DC (as well as spontaneous elimination of DC, Bjorck *et al.*, 1997; Koppi *et al.*, 1997) it is plausible that *in vivo* mature DC survive when encountering properly activated specific CD40L$^+$ T cells, but die in the presence of not properly activated CD40L-defective T cells (namely anergic T cells). Of interest, DC acquire susceptibility to the active pathway of apoptosis (CD95/CD95L-mediated) after full maturation. Indeed, in our hands, DC cultured for 6 days with GM-CSF and IL-4 did not express detectable levels of CD95, but in 3 more days of culture up-regulated CD95 and became susceptible to apoptosis (M.M.). This renders DC that have reached complete maturation still sensitive to suppressive stimuli of anergic/suppressor cells that can regulate their functions at different moments of their life-span.

Another very relevant finding is that anergic cells can also determine death of responder T cells, but these latter are eliminated only if responder and anergic T cells recognize antigen on the same APC (Fig. 3b). This ensures suppression to work as an antigen specific phenomenon. Nevertheless, it remains to be clarified if all responder cells die and, if not, what the fate is of these surviving cells. In other words, if "suppressed" cells (included T cells suppressed in the presence of immature DC) are, in turn, rendered tolerant or deleted by other means. Finally, the low amount of CD40L on anergic cells fails to induce up-regulation of an anti-apoptotic molecule such as Bcl-2 that may inhibit also spontaneous apoptotic of DC, a phenomenon that we observed following 24–48 h of co-culture (not shown in this paper). Regarding this latter point, although the mechanism of active death seems to be the most relevant for T cell suppression (we could significantly abrogate suppression by using the antagonistic anti-CD95 Ab in suppression experiments, Fig. 3a) limitation of the half-life of DC available for primary stimulation of other helper T cells or effector cells (such as CD8 T cells) can also represent a crucial parameter in the induction of cellular immune responses (de Smedt *et al.*, 1998; Miga *et al.*, 2001).

### MATERIAL AND METHODS

### Reagents, T Cell Lines, Clones

Peptide HA307-19 was synthesized by F-MOC chemistry (Frasca *et al.*, 1997). Antibodies used were: OKT3 (anti-human CD3, ATCC, Rockville, MD); antagonistic anti-CD95 (M3, Immunex, Seattle, WA); anti-mouse IgG, for cross-linking of CD40 mAb (Sigma Chemical Co., St. Louis, MO); L243 (IgG2a, anti-DR, ATCC); R-PE-conjugated anti-CD154 (CD40L), (TRAP-1, BD PharMingen, San Diego, CA). FITC-conjugated hamster anti-Bcl-2 (BD PharMingen). Anti CD86, Bu63, kindly provided by Peter Beverly, London, UK. Anti-CD40L (blocking) 24-31 (Alexis, Biochemicals, Vinci-Biochem, FI, Italy). T cell clones F17, HA307-19-specific and DRB1*1101-restricted, was derived from a DRB1*0101/DRB1*1101 individual. The homozigous lymphoblastoid cell line (B-LCL) used as APC was Sweig (DRB1*1101, DQA1*0501, DQB1*0301).

### Generation, Culture and Phenotypical Characterization of Human DC

DC were prepared from PBMC of healthy donors. Adherent cells were cultured in RPMI 2% HS plus 50 ng/ml of GM-CSF and 100 u/ml rIL-4 (Roche Molecular Biochemicals, Mannheim, Germany). After 6 days of culture $CD1a^+$, $CD14^-$, mannose $receptor^+$, HLA-class $II^+$ and $CD4^+$ DC were defined "immature DC". "Immature DC" reached spontaneous maturation by up-regulating CD40, HLA-DR, CD86 and CD95 during a prolonged culture of at least 3 days ("mature DC"). CD86, HLA-DR and Bcl-2 expression on DC co-cultured T cells was assessed by staining with Bu63, L243 and anti-Bcl-2 Abs, respectively. $10^4$ "immature" DC were cultured for 24 or 48 h either alone or in the presence of $10^5$ T cells in flat bottom 96-plates in RPMI 5% HS. Bcl-2 expression was tested on DC fixed with PBS 1 × 2% paraformaldeide, washed in PBS 1 × 0.5 BSA, 0.02% sodium azide and permeabilized in PBS 1 × 0.5% BSA, 0.02% sodium azide, 0.5% saponine. Isotype matched mAbs were always included and cells analyzed by Beckton Dickinson FACScalibur flow cytometer (FACS).

### Anergy Induction, T Cell Suppression Assay and Cytotoxic Assay

T cell anergy was obtained as previously described by incubating T cells overnight (o.n.) with immobilized mAb anti-CD3 (OKT3). Lack of proliferation and IL-2 production, was tested in proliferation and CTLL-2 assay, respectively (Frasca *et al.*, 1997). The cells that did not proliferate and did not synthesize IL-2 were considered "anergic". For suppression experiments T cells ($5 \times 10^3$) were cultured with $5 \times 10^3$ irradiated either "immature" or "mature" DC in flat bottom microtiter plates in a total volume of 200 μl in RPMI 1640 (Gibco BRL, Paisley, Scotland), 10% HS, plus/minus $1.5 \times 10^4$ anergic T cells. In some experiments responder T cells were also cultured with DC in the presence of T cells activated with 0.05 μM PMA (Sigma Chemical Co.) + 0.5 μM Ionomycin (Calbiochem, La Jolla, CA) for 4 h. After 48 h wells were pulsed with 1 μCi of $^3$H-TdR (Amersham International, Amersham, UK) and harvested onto glass fiber filters 18 h later. Proliferation was measured as $^3$H-TdR incorporation by liquid scintillation spectroscopy. The results are expressed as mean counts of triplicate cultures. Standard errors were routinely < 10%. Lytic activity of anergic T cells (effector cells) on target DC or responder T cells was assessed by standard $^{51}$Cr-release assay. Target cells $3 \times 10^3$, labeled with $^{51}$Cr for 1 h, were cultured in a 96-well round-bottom plate with $9 \times 10^3$ effector cells (effector/target ratio 3:1). Untreated responder T cells were also used as control effectors and their killing activity was always < 10%. After 4 h, $^{51}$Cr release in the supernatants was determined on a ME Plus γ-scintillation counter (Micromedic Systems, Huntsville, TN). Specific lysis was calculated as 100 × (experimental release—spontaneous release)/(maximum release—spontaneous release).

### References

Bjorck, P., Banchereau, J. and Flores-Romo, L. (1997) "CD40 ligation counteracts CD95-induced apoptosis of human dendritic cells", *Int. Immunol.* **9**, 365–372.

Bowen, F., Haluskey, J. and Quill, H. (1995) "Altered CD40 ligand in tolerant lymphocytes", *Eur. J. Immunol.* **25**, 2830–2834.

Charlton, B., Auchincloss, H. Jr. and Fathman, G.C. (1994) "Mechanisms of transplantation tolerance", *Annu. Rev. Immunol.* **12**, 707–734.

de Smedt, T., Pajak, B., Klaus, G.G.B., Noelle, R.J., Urbain, J., Leo, O. and Moser, M. (1998) "Antigen specific T lymphocytes regulate lipopolysaccharide-induced apoptosis of dendritic cells *in vivo*", *J. Immunol.* **161**, 4476–4479.

Frasca, L., Carmichael, P., Lechler, R.I. and Lombardi, G. (1997) "Anergic T cells affect linked suppression", *Eur. J. Immunol.* **27**, 3191–3197.

Gershon, R.K. (1975) "A disquisition on suppressor T cells", *Transplant. Rev.* **26**, 170–185.

Koppi, T.A., Tough-Bement, T., Lewinsohn, D.M., Lynch, D.H. and Alderson, R. (1997) "CD40 ligand inhibits CD95/CD95-mediated apoptosis of human blood-derived dendritic cells", *Eur. J. Immunol.* **27**, 3161–3165.

Lanzavecchia, A. (1998) "Licence to kill", *Nature* **393**, 413–414.

Lombardi, G., Sidhu, S., Batchelor, R. and Lechler, R.I. (1994) "Anergic T cells as suppressor cells *in vitro*", *Science* **264**, 1587–1589.

Ludewig, B., Graf, D., Gelderblom, H.R., Becker, Y., Kroczek, R.A. and Pauli, G. (1995) "Spontaneous apoptosis of dendritic cells is efficiently inhibited by TRAP (CD40-ligand) and TNF-α, but strongly enhanced by interleukin-10", *Eur. J. Immunol.* **25**, 1943–1950.

Miga, A.J., Master, S.R., Durell, B.G., Gonzales, M., Jenkins, M.K., Maliszewski, C., Kikutani, H., Wade, W.F. and Noelle, R.J. (2001) "Dendritic cell longevity and T cell persistence is controlled by CD154–CD40 interactions", *Eur. J. Immunol.* **31**, 959–965.

Vendetti, S., Chai, J.G., Dyson, J., Simpson, E., Lombardi, G. and Lechler, R.I. (2000) "Anergic T cells inhibit the antigen-presenting function of dendritic cells", *J. Immunol.* **165**, 1175–1182.

Zhai, Y. and Kupie-Weglinski, J.W. (1999) "What is the role of regulatory T cells in transplantation tolerance?", *Curr. Opin. Immunol.* **11**, 497–503.

